# PBK phosphorylates MSL1 to elicit epigenetic modulation of *CD276* in nasopharyngeal carcinoma

**DOI:** 10.1038/s41389-020-00293-9

**Published:** 2021-01-05

**Authors:** Meng-Yao Wang, Bin Qi, Fang Wang, Zhi-Rui Lin, Ming-Yi Li, Wen-Jing Yin, Yan-Yi Zhu, Lu He, Yi Yu, Fang Yang, Jin-Quan Liu, Dong-Ping Chen

**Affiliations:** 1 Department of Radiation Oncology, Affiliated Cancer Hospital and Institute of Guangzhou Medical University, 510245 Guangzhou, China; 2 State Key Laboratory of Oncology in South China, Collaborative Innovation Center for Cancer Medicine, Sun Yat-Sen University Cancer Center, 510245 Guangzhou, China

**Keywords:** Head and neck cancer, Tumour immunology

## Abstract

CD276 (also known as B7–H3, an immune checkpoint molecule) is aberrantly overexpressed in many cancers. However, the upregulation mechanism and in particular, whether oncogenic signaling has a role, is unclear. Here we demonstrate that a pro-oncogenic kinase PBK, the expression of which is associated with immune infiltration in nasopharyngeal carcinoma (NPC), stimulates the expression of *CD276* epigenetically. Mechanistically, PBK phosphorylates MSL1 and enhances the interaction between MSL1 and MSL2, MSL3, and KAT8, the components of the MSL complex. As a consequence, PBK promotes the enrichment of MSL complex on *CD276* promoter, leading to the increased histone H4 K16 acetylation and the activation of *CD276* transcription. In addition, we show that *CD276* is highly upregulated and associated with immune infiltrating levels in NPC. Collectively, our findings describe a novel PBK/MSL1/CD276 signaling axis, which may play an important role in immune evasion of NPC and may be targeted for cancer immunotherapy.

## Introduction

Nasopharyngeal carcinoma (NPC) is one of the most common head and neck cancers in southeast Asia and north Africa^1,2^. The standard treatment for patients with NPC is concurrent chemoradiation preceded or followed by systemic chemotherapy, according to the National Comprehensive Cancer Network (NCCN) guidelines. Although the local control rate has been significantly improved, approximately 30–40% of patients with locoregionally advanced NPC eventually develop distant metastasis after receiving radical treatment^3^. Novel strategies are alarmingly needed for NPC patients with a high risk of distant metastasis.

Evading immune destruction is described as the hallmark of cancer^4^. NPC originated from the epithelium of the nasopharyngeal, which is regarded as a highly immunogenic tumor characterized by heavy tumor-infiltrating lymphocytes (TILs), therefore at one time called “lymphoepithelial neoplasia”^5^. Furthermore, Epstein–Barr virus (EBV) infection is a major risk factor for the development of NPC in the endemic regions^6,7^, upregulation of programmed cell death-ligand 1 (PDL1) was found in EBV-driven NPC cells, it is reported that PDL1 (CD274) expression on NPC is associated with a poor outcome^8^, this suggests that the development of NPC may be closely related to the immune escape of tumor cells. In pursuit of the novel effective treatment, several single-arm trials investigate that PD1 (CD279) inhibitors are effective in only 20–30% of recurrent or metastatic NPC patients^9,10^, it is urgent to develop more biomarkers to reconstruct the immune surveillance, which would be exceedingly beneficial for the clinical intervention.

CD276, also known as B7–H3, is a type I membrane protein with its sequence similarity to the extracellular domain of other B7 family members, which modulate T-cell function in a co-stimulatory or co-inhibitory manner^11–13^. The CD276 protein is rarely expressed and is only found at low levels in normal human tissues^14,15^. By contrast, recent studies found aberrant high CD276 expression on the many common malignancies, including stomach^16^, lung^17^, prostate^18^, kidney^19^, ovary^20^, and endometrium^21^, and the high expression of CD276 was also correlated with advanced disease and poor outcome^22^. Moreover, CD276 protein is also frequently overexpressed on tumor vessels of human lung, breast, colon, endometrial, renal, and ovarian cancer, but not in the angiogenic vessels of the normal ovary^15^. Notably, an increasing number of studies support a pro-oncogenic role for CD276 in various human cancer types that is independent of its immune function^15,23,24^. Overexpression of CD276 makes it an attractive target for the development of therapeutic agents, and in fact, CD276-targeting therapies are currently under clinical investigation in several children and adult tumors (NCT02982941, NCT01391143, NCT01099644, NCT02381314, and NCT04185038)^15,25–31^. However, the cellular mechanisms that promote *CD276* expression are poorly understood.

A PDZ-binding kinase (PBK), also known as lymphokine-activated killer T-cell-originated protein kinase (TOPK), is a member of the novel MEK3/6-related mitogen-activated protein kinase–kinase (MAPKK) family^32^ and directly phosphorylates ERK, H2AX (Ser139), peroxiredoxin 1 (PRX1, Ser32), JNK1 (Thr183/Tyr185), and p53-related protein kinase (PRPK, Ser250)^33–35^. Thus, PBK activates downstream signaling cascades via its phosphorylated substrates and plays an important role in many kinds of cellular processes, including growth, development, apoptosis, and inflammation^36^. Previous studies including our group have highlighted that PBK was required for malignant phenotypes and was associated with poor prognosis of human common cancers, including NPC, oral cancer, breast cancer, colorectal cancer, leukemia and lymphoma, ovarian cancer, lung cancer, and glioma^34,36–40^. Due to the broad expression of PBK across multiple tumor types, specific inhibitors have been developed to target PBK, including HI-TOPK-032^41^, OTS514/OTS964^42^, and ADA-07^43^, and show high therapeutic potency in the preclinical study. PBK has been revealed to contribute to the regulation of proliferation and cell cycle progression, whereas whether PBK involves in the tumor immune evasion is not reported so far. In the current study, we revealed that PBK promotes the transcription of *CD276* via an epigenetic way, providing new insight into immune evasion in NPC.

## Results

### *PBK* expression is associated with immune infiltration

**Fig. 1:**
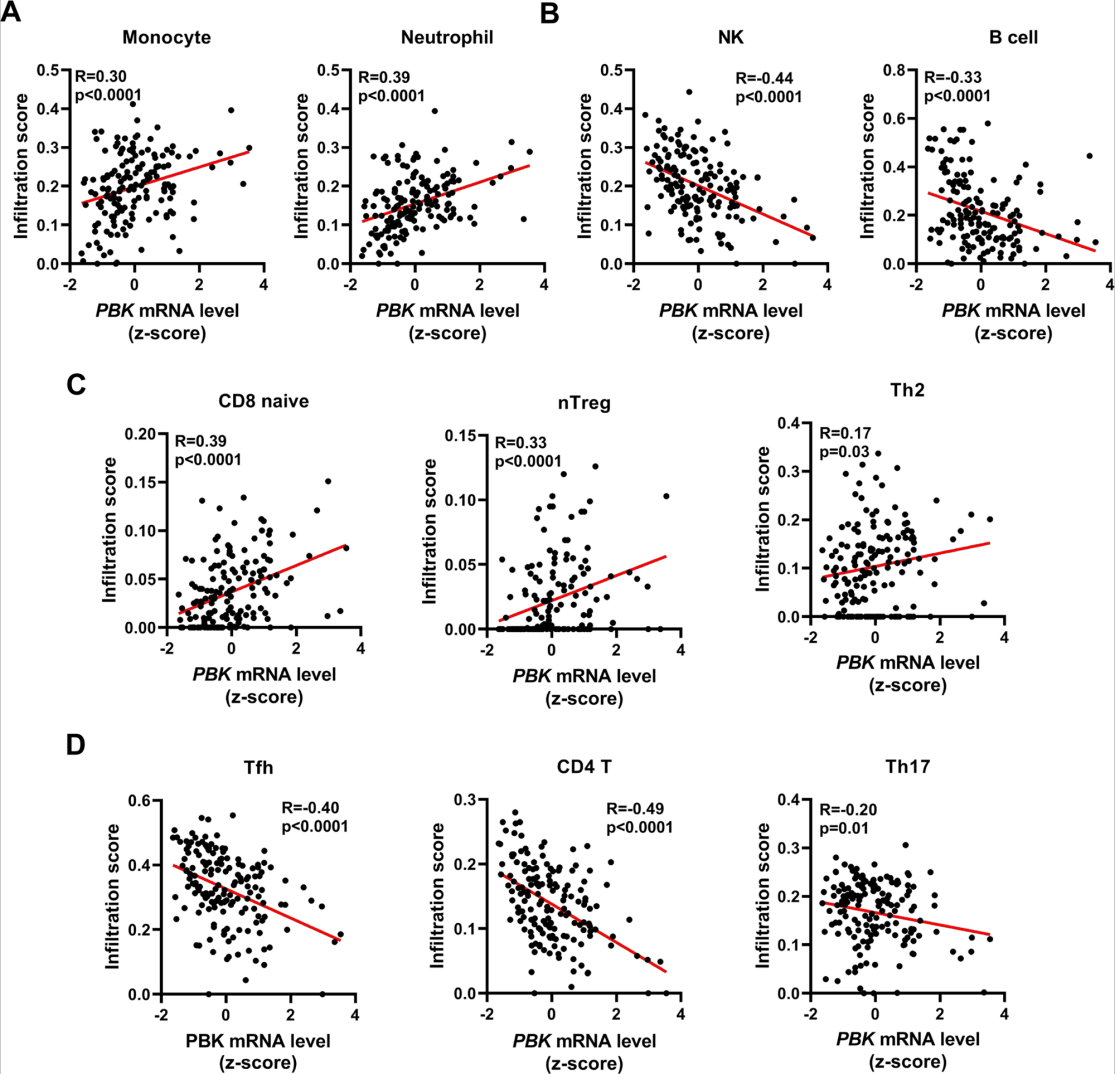
*PBK* mRNA level is associated with immune cell infiltration. **A** The scatterplots show that the mRNA level of *PBK* (lymphokine-activated killer T-cell-originated protein kinase) is significantly correlated with the infiltration of neutrophil cells (left), monocyte cells in NPC (nasopharyngeal carcinoma). **B** The scatterplots show that the mRNA level of *PBK* is negatively correlated with the infiltration of NK (natural killer) cells (left) and B cells in NPC. **C** The scatterplots show that the mRNA level of *PBK* is significantly correlated with the infiltration of CD8-naive cell (left), nTreg cell (middle), and Th2 cell. **D** The scatterplots show that the mRNA level of *PBK* is negatively correlated with the infiltration of Tfh cell (left), CD4+ T cell (middle), and Th17 cell. The method called ImmuCellAI was used to estimate the abundance of 24 immune cell types from two integrated NPC RNA-seq data (GSE102349, GSE68799). A Pearson correlation test was used (two-tailed) (*n* = 159).

The level of TILs is an independent predictor of sentinel lymph node status and survival in many cancers including NPC^44^. To explore the role of *PBK* in immune regulation of NPC, we investigated whether *PBK* expression was correlated with immune infiltration levels in NPC. ImmuCellAI^45^, a highly accurate method, was used to estimate the infiltration abundance of immune cells from RNA-seq expression profiles of two NPC cohorts (GSE102349 and GSE68799). Interestingly, correlation analysis showed that *PBK* expression was significantly correlated to the infiltration of neutrophil and monocyte cells (Fig. 1a), which were reported to promote the immune evasion of tumors^46–48^, but negatively correlated to the infiltration of natural killer (NK) and B cells (Fig. 1b), which were reported to repress the immune evasion of tumors^49–51^. Moreover, ImmuCellAI was designed to estimate the abundance of 18 T-cell subsets that are major players in the tumor microenvironment. Notably, *PBK* expression has significant correlations with CD8-naive cell, nTreg cell, and Th2 cell (Fig. 1c), but was negatively correlated to the infiltration of Tfh cell, CD4+ T cell, and Th17 cell (Fig. 1d). Based on these results, it is supposed that high *PBK* expression is closely associated with compromised immune microenvironment in NPC.

### PBK regulates the transcription of *CD276*

**Fig. 2:**
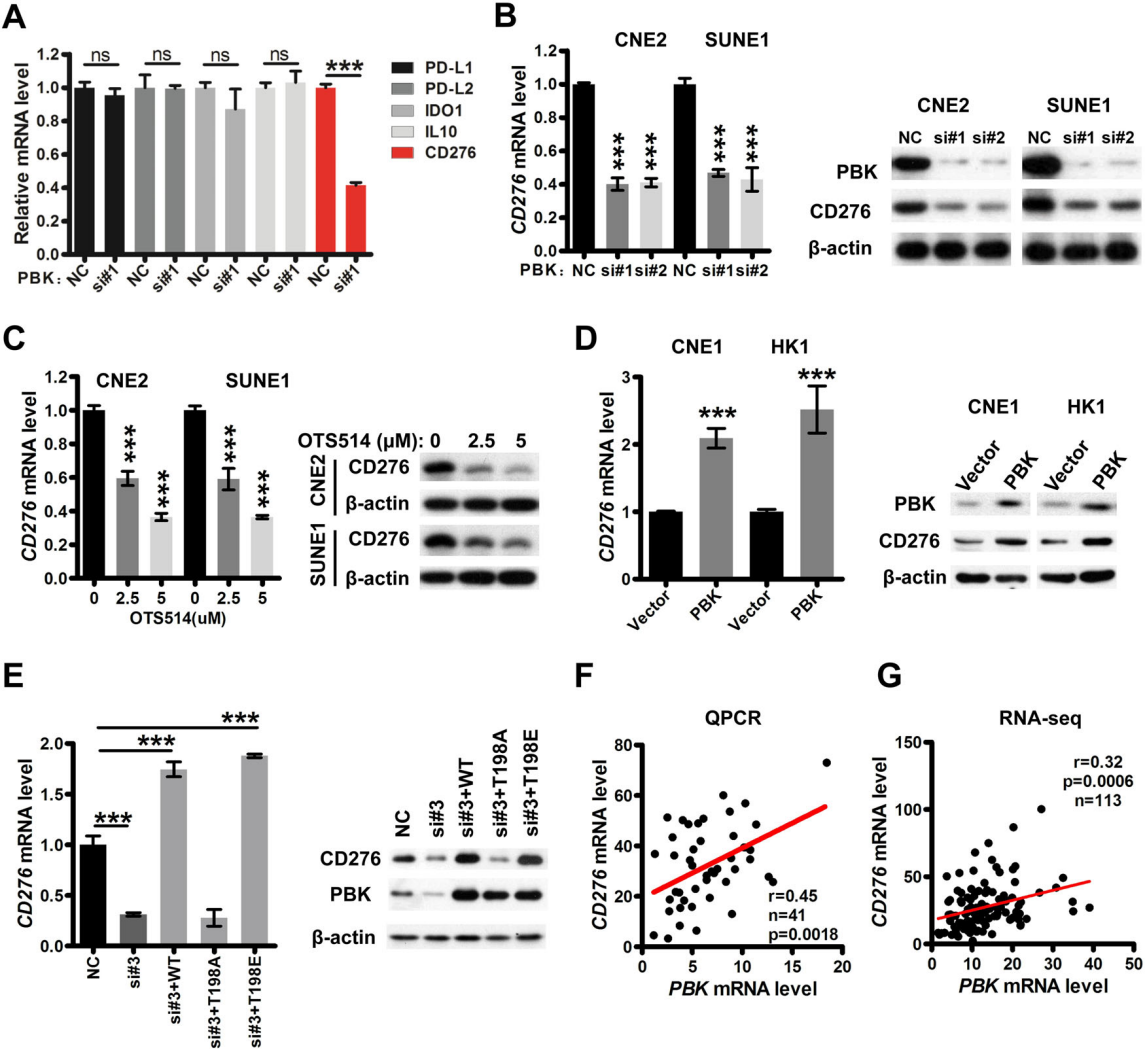
PBK regulates the transcription of *CD276* dependent on its kinase activity. **A** CNE2 cells were transfected with one *PBK* siRNA or NC (nonspecific siRNA) for 48 h and qPCR (quantitative polymerase chain reaction) was performed to measure the mRNA expression of indicated genes. **B** CNE2 and SUNE1 cells were transfected with two *PBK* siRNAs or NC (nonspecific siRNA) for 48 h. qPCR and immunoblotting were conducted to measure the mRNA and protein expression of *CD276*. Left, qPCR. Right, immunoblotting with indicated antibodies. **C** CNE2 and SUNE1 cells were treated with DMSO (dimethylsulfoxide) or OTS514 (2.5 and 5 μM) for 24 h. qPCR and immunoblotting were conducted to measure the mRNA and protein expression of *CD276*. Left, qPCR. Right, immunoblotting with indicated antibodies. **D** CNE1 and HK1 cells were infected by lentiviruses carrying *PBK* coding region to establish the PBK-overexpression stable cell lines. qPCR and immunoblotting were conducted to measure the mRNA and protein expression of *CD276*. Left, qPCR. Right, immunoblotting with indicated antibodies. **E** CNE2 and SUNE1 cells were transfected with one siRNA targeting PBK 3′UTR (untranslated region) region or NC (nonspecific siRNA). After 24 h, the cells were transfected with *PBK* constructs, including WT (wild type), kinase-dead (T198A), or kinase consistently activated (T198E) for another 24 h. qPCR and immunoblotting were conducted to measure the mRNA and protein expression of *CD276*. Left, qPCR. Right, immunoblotting with indicated antibodies. **A**–**E** In qPCR assays, *ACTB* was used as the loading control, and the mRNA level of indicated genes is normalized to *ACTB*. The results are shown as the fold change over NC (nonspecific siRNA). Data present as the mean ± SEM (standard error of the mean) (*n* = 3). ***P < 0.001 (student’s *t* test for two groups, one-way ANOVA for three groups). All the experiments were performed independently three times with similar results, and the data are representative of three independent experiments. **F**–**G** Correlation analyses between the *PBK* and *CD276* mRNA level were performed using our qPCR data (**F**) or public RNA-seq data (**G**). A Pearson correlation test was used (two-tailed).

Given the pivotal roles of T-cell subsets in cancer initiation, progression, and immunotherapy^52–54^, we focus on how PBK modulates the infiltration of T-cell subsets. Accumulating evidence has indicated that cancer cells could upregulate a series of immune inhibitory factors to repress the proliferation, cytokine production, and effector activities of T cells, in turn leading to immune evasion^55–57^. Thus, we investigated whether previously reported immune inhibitory molecules of T cells, including *PDL1 (CD274)*, *PDL2 (CD273)*, *CD276*, *IL10*, and *IDO1*, could be downstream targets of PBK. The results of quantitative polymerase chain reaction (qPCR) showed that only *CD276* was significantly downregulated in CNE2 cells after PBK knockdown by siRNA in our primary screening (Fig. 2a and Fig. S1). Further, qPCR and immunoblotting assays revealed that the mRNA and protein level of *CD276* were downregulated in CNE2 and SUNE1 cells with PBK depletion by two siRNAs (Fig. 2b and Fig. S2a), but were upregulated in HK1 and CNE1 cells with PBK overexpression (Fig. 2c and Fig. S2b), suggesting that *CD276* is a bona fide downstream target of PBK.

To investigate whether the PBK kinase activity is indispensable for the regulation of *CD276*, we treated CNE2 and SUNE1 cells with OTS514, a PBK kinase-specific inhibitor^58,59^, followed by qPCR and immunoblotting assays, and found that compared to DMSO treatment, OTS514 treatment decreased the mRNA and protein level of CD276 in a dose-dependent manner (Fig. 2d and Fig. S2c). According to previous reports^39^, we construct two PBK mutants, including kinase-active mutants (PBK-T198E) and kinase-dead mutants (PBK-T198A). Both qPCR and immunoblotting assays revealed that PBK-WT and PBK-T198E but not PBK-T198A could rescue the repression of *CD276* mediated by a PBK siRNA that targets the *3*′*UTR* region (Fig. 2e and Fig. S2d). These data show that PBK regulates *CD276* transcription dependent on its kinase activity.

Furthermore, correlation analysis showed that the mRNA expression of *PBK* and *CD276* is positively correlated in our NPC cohort detected by qPCR (*r* = 0.45, *n* = 41, *p* = 0.0018, Fig. 2f) as well as in the NPC RNA-seq dataset (*r* = 0.32, *n* = 113, *p* = 0.0006, Fig. 2g). Notably, pan-cancer correlation analysis between *PBK* and *CD276* was conducted using the mRNA expression data from TCGA. The result reveals that the mRNA level of *PBK* is positively correlated with *CD276* in several common cancers (Fig. S3), including adrenocortical carcinoma, brain lower-grade glioma, kidney renal clear-cell carcinoma, kidney renal papillary cell carcinoma, liver hepatocellular carcinoma, lung adenocarcinoma, pancreatic adenocarcinoma, prostate adenocarcinoma, and thyroid carcinoma, indicating that this is a general mechanistic upregulation of *CD276* by PBK in cancers.

### PBK regulates *CD276* transcription dependent on MSL1

**Fig. 3:**
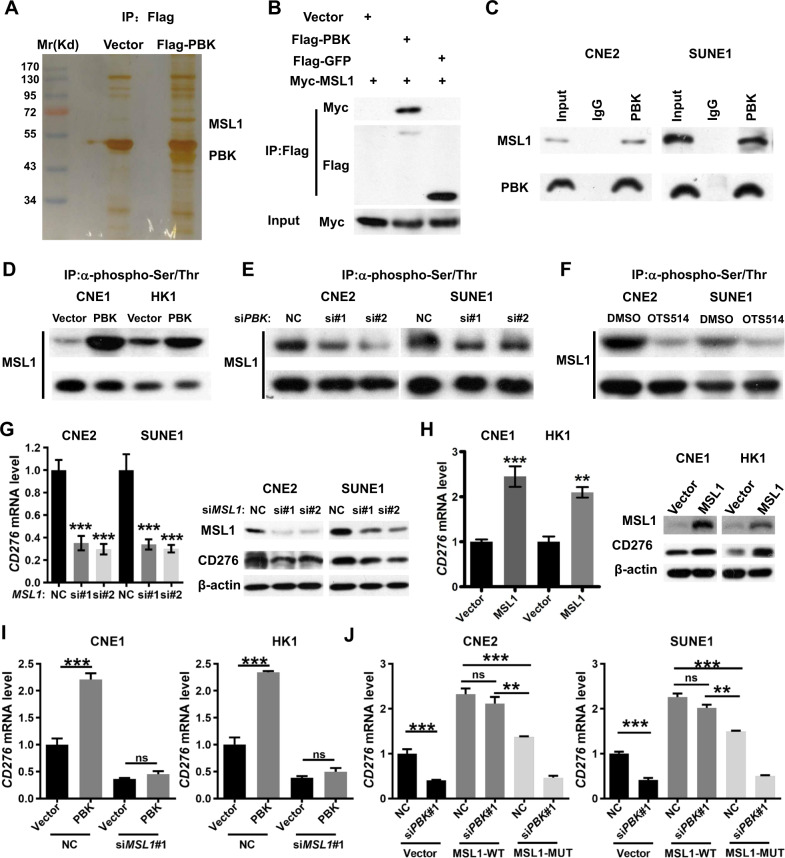
PBK regulates the expression of *CD276* dependent on MSL1. **A** SDS/PAGE and silver staining analysis of proteins pulled down by Flag-PBK or vector, via anti-Flag affinity purification. Mass spectrometry analysis recognized MSL1 (male-specific lethal 1 homolog) as an interacting partner of PBK. **B** 293T cells were co-transfected Myc-MSL1 with vector, Flag-GFP (green fluorescent protein), or Flag-PBK for 36 h, and immunoprecipitation assays and immunoblotting analyses with the indicated antibodies were performed. **C** Endogenous immunoprecipitation assay in CNE2 and SUNE1 cells with an anti-PBK antibody followed by immunoblotting with antibodies against the indicated proteins. **D** Immunoprecipitation assays and immunoblotting analyses with the indicated antibodies were performed in PBK-overexpression stable cell lines or control cell lines. **E** CNE2 and SUNE1 cells were transfected with *PBK* siRNAs or NC (nonspecific siRNA) for 48 h. Immunoprecipitation assays and immunoblotting analyses with the indicated antibodies were performed. **F** CNE2 and SUNE1 cells were treated with DMSO or OTS514 (5 μM) for 24 h. Immunoprecipitation assays and immunoblotting analyses with the indicated antibodies were performed. **G** CNE2 and SUNE1 cells were transfected with two *MSL1* siRNAs or NC (nonspecific siRNA) for 48 h. qPCR and Immunoblotting were conducted to measure the mRNA and protein expression of *CD276*. Left, qPCR. Right, immunoblotting with indicated antibodies. **H** CNE1 and HK1 cells were infected by lentiviruses carrying *MSL1* coding region to establish the MSL1 overexpression stable cell lines. qPCR and immunoblotting were conducted to measure the mRNA and protein expression of *CD276*. Left, qPCR. Right, immunoblotting with indicated antibodies. **I** PBK-overexpression cell lines or control cell lines were transfected with one *MSL1* siRNA or NC (nonspecific siRNA) for 48 h, respectively. qPCR assays were conducted to measure the mRNA level of *CD276*. Left, CNE1 cell lines, right, HK1 cell lines. **J** MSL1 wild type or serine/threonine residues' mutant overexpression cell lines or control cell lines were transfected with a *PBK* siRNA or NC (nonspecific siRNA) for 48 h. qPCR assays were conducted to measure the mRNA level of *CD276*. Left, CNE2 cell lines, right, SUNE1 cell lines. **J** In qPCR assays, *ACTB* was used as the loading control, and the mRNA level of *CD276* is normalized to *ACTB*. The results are shown as the fold change over NC (nonspecific siRNA). Data present as the mean ± SEM (*n* = 3). **P < 0.01, ***P < 0.001, ns not significant (Student’s *t* test for two groups, one-way ANOVA for three groups). All the experiments were performed independently three times with similar results and the data are representative of three independent experiments.

Considering that PBK is a kinase, not a transcription regulator, there is a mediator involved in *CD276* transcription regulation by PBK. In order to identify the mediator, we carried out immunoprecipitation and mass spectrometry analysis and identified MSL1 as an interacting partner of PBK (Fig. 3a). Co-immunoprecipitation assays validated the exogenous interaction between PBK and MSL1 in 293T cells (Fig. 3b). To further confirm the in vivo interaction, total proteins from CNE2 and SUNE1 cells were extracted, and immunoprecipitation was performed with an antibody against PBK followed by immunoblotting with an antibody against MSL1, demonstrating that MSL1 was efficiently co-immunoprecipitated with PBK (Fig. 3c). Since PBK has serine/threonine kinase activity, we investigated whether MSL1 is a phosphorylation target of PBK. Immunoprecipitation assays using an antibody against phosphorylated serine/threonine followed by immunoblotting analysis using MSL1 antibody demonstrated that the phosphorylation level of MSL1 was enhanced in PBK-overexpression cells (Fig. 3d and Fig. S4a), but was decreased in PBK-knockdown cells (Fig. 3e and Fig. S4b) as well as in cells treated with PBK inhibitor (Fig. 3f and Fig. S4c). These data show that PBK indeed interacts with and phosphorylates MSL1.

Furthermore, qPCR and immunoblotting results showed that MSL1 depletion decreased the expression of *CD276* (Fig. 3g and Fig. S4d) and MSL1 overexpression increased the expression of *CD276* (Fig. 3h and Fig. S4e), suggesting that MSL1 is involved in the transcriptional regulation of *CD276*. More importantly, the knockdown of MSL1 repressed the upregulation of *CD276* mediated by PBK overexpression (Fig. 3i and Fig. S4f). MSL1 contains multiple serine/threonine residues, six of which (Ser66, Ser126, Ser205, Ser393, Thr396, and Ser442) were reported as phosphorylation sites^60,61^. Given that the target sites of MSL1 phosphorylated by PBK are unknown, we replaced all of those MSL1 serine/threonine residues noted above individually with an alanine residue (S/T > A-MSL1). The result of qPCR showed that MSL1 wild-type but not MSL1-S/T > A mutant overexpression could rescue the downregulation of *CD276* after PBK depletion in CNE2 and SUNE1 cells (Fig. 3j and Fig. S4g), indicating that PBK controls the transcription of *CD276* dependent on the phosphorylation status of MSL1.

### PBK promotes the enrichment of MSL complex on *CD276* promoter

**Fig. 4:**
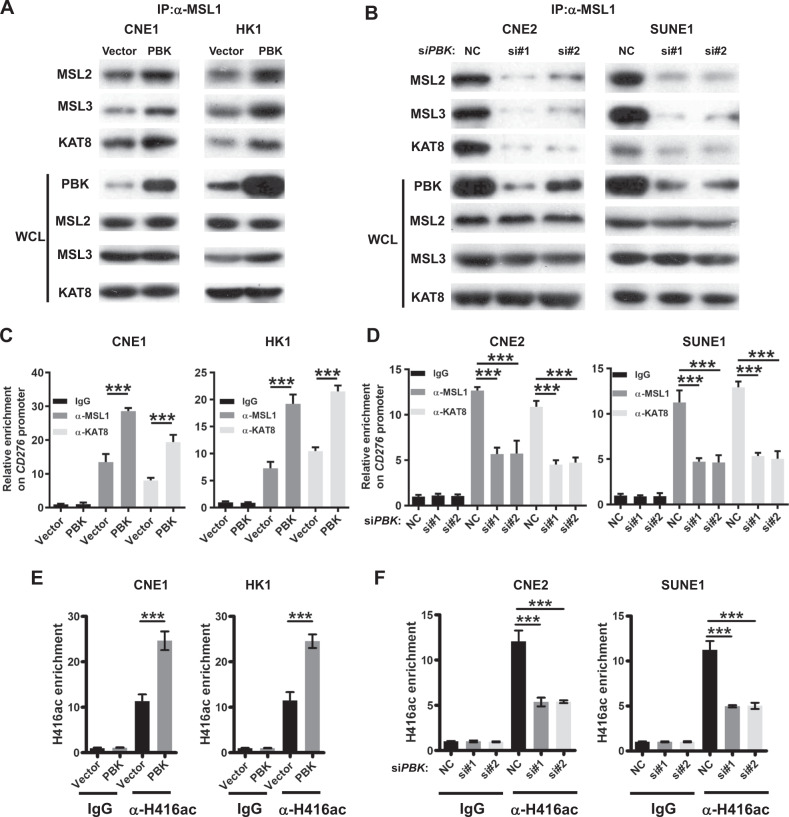
PBK promotes the formation of MSL complex on the promoter of *CD276*. **A** Immunoprecipitation assays with MSL1 antibody and immunoblotting analyses with the indicated antibodies were performed in PBK-overexpression stable cell lines. Left, CNE1, right, HK1. **B** CNE2 and SUNE1 cells were transfected with *PBK* siRNAs or NC (nonspecific siRNA) for 48 h. Immunoprecipitation assays with MSL1 antibody and immunoblotting analyses with the indicated antibodies were performed. Left, CNE2, right, SUNE1. **C** ChIP-qPCR (chromatin immunoprecipitation-quantitative PCR) analysis of *CD276* promoter using antibodies against MSL1 and KAT8 in PBK-overexpression or control cell lines. Left, CNE1, right, HK1. **D** ChIP-qPCR analysis of *CD276* promoter using antibodies against MSL1 and MOF in PBK-knockdown or control cells. Left, CNE2, right, SUNE1. **E**, **F** ChIP-qPCR analysis of *CD276* promoter using antibodies against H4K16ac (acetylation of histone H4 on lysine 16) in PBK-overexpression (**E**) or -knockdown cells (**F**). In **C**–**F**, ChIP-qPCR results were presented as the fold change over the vector/IgG or NC/IgG group. Error bars represent the mean ± SD (standard deviation) for three biological replicates. ***P < 0.001 (student’s *t* test for two groups, one-way ANOVA for three groups). All the experiments were performed independently three times with similar results and the data are representative of three independent experiments.

MSL1 acts as a scaffold to tether MSL2, MSL3, and KAT8 (MOF) together for the formation of the MSL histone acetyltransferase complex, in turn leading to histone H4 Lys16 acetylation (forming H4K16ac), thereby efficiently increasing the gene expression^62,63^. Apart from the dosage-compensation role of the MSL complex, accumulating evidence suggests that MSL proteins may be involved in additional functions, such as transcription regulation^64–67^. To address the role of PBK in the MSL complex, we conducted immunoprecipitation experiments using an antibody against MSL1 followed by immunoblotting with antibodies against the components of the MSL complex in PBK-overexpression or PBK-knockdown cells, respectively. The results demonstrated that PBK overexpression could enhance the physical association between MSL1 and MSL2, MSL3, and KAT8 (Fig. 4a and Fig. S5a), whereas PBK depletion obviously inhibited these interactions (Fig. 4b and Fig. S5b). Moreover, quantitative chromatin immunoprecipitation (qChIP) analysis using specific antibodies against MSL1 or KAT8 showed that the occupancy of MSL1 and KAT8 on the *CD276* promoter region is increased in PBK-overexpression cells (Fig. 4c) but decreased in PBK- deficient cells (Fig. 4d). Consistently, the acetylation level of histone H4 Lys16 on the promoter region is increased in PBK-overexpression cells but decreased in PBK-deficient cells, as indicated by qChIP results (Fig. 4e, f).

### High expression of *CD276* is associated with the immune infiltration in NPC

**Fig. 5:**
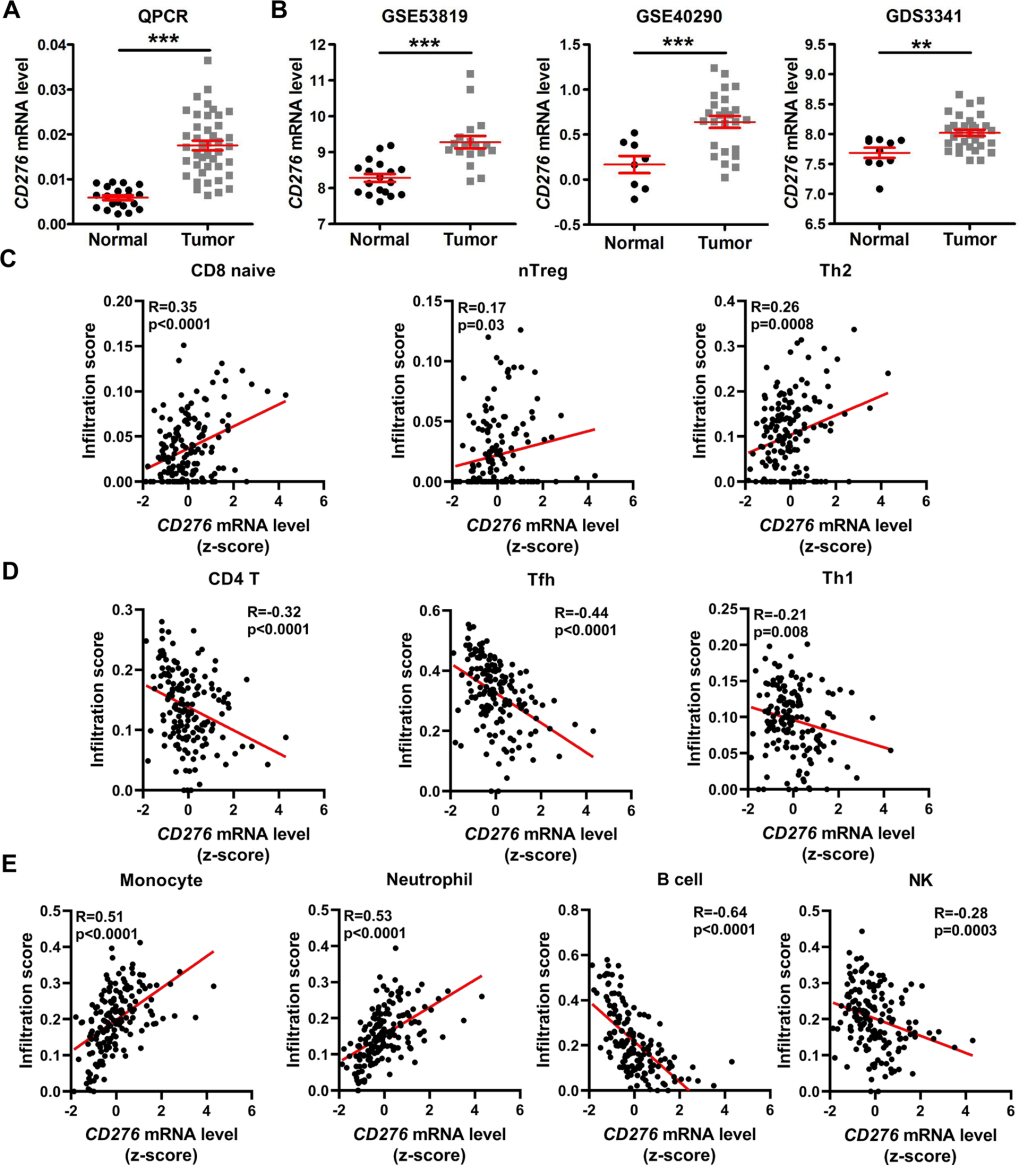
*CD276* is upregulated and associated with immune infiltration in NPC. **A** The graphs show that *CD276* is significantly upregulated in NPC samples (*n* = 41) compared to normal samples (*n* = 20) via qPCR analysis. ***P < 0.001 was determined by Student’s *t* test. **B** The graphs show that *CD276* is significantly upregulated in NPC samples compared to normal samples, as indicated by four public datasets from GEO (Gene Expression Omnibus database). **P < 0.01, ***P < 0.001 was determined by Student’s *t* test. **C** Correlation analysis between the *CD276* mRNA level and the tumor-promotion T-cell subset infiltration, including CD8-naive cell (left), nTreg cell (middle), and Th2 cell using publicly available NPC RNA-seq data. A Pearson correlation test was used (two-tailed). **D** Correlation analysis between the *CD276* mRNA level and the tumor-repression T-cell subset infiltration, including CD4 T cell (left), Tfh cell (middle), and Th1 cell using publicly available NPC RNA-seq data. A Pearson correlation test was used (two-tailed). **E** Correlation analysis between the *CD276* mRNA level and the infiltration level of monocyte, neutrophil, NK, and B cells using publicly available NPC RNA-seq data. A Pearson correlation test was used (two-tailed).

Since the role of *CD276* in NPC remains unknown, we performed a qPCR assay and found that *CD276* mRNA expression in NPC tissues was higher than in the noncancerous NP tissues (Fig. 5a). In addition, in agreement with our qPCR result, gene expression profiles derived from the GEO (GDS3341, GSE40290, and GSE53819^68^) showed that compared to noncancerous NP tissues, the *CD276* mRNA level was obviously elevated in the NPC tissues (Fig. 5b). Previous reports showed that CD276 has been associated with co-stimulatory as well as co-inhibitory functions in regulating T-cell responses^12,14,69,70^. In NPC, correlation analysis showed that *CD276* mRNA level was positively associated with the infiltration level of CD8-naive cell, nTreg cell, and Th2 cell (Fig. 5c), but was negatively associated with the infiltration level of CD4 T cell, Tfh cell, and Th1 cell (Fig. 5d). In addition, *CD276* expression has a significant correlation with monocyte and neutrophil cells and a negative correlation with NK and B cells (Fig. 5e). Taken together, our findings demonstrated that *CD276* is upregulated in NPC and associated with immune cell infiltration.

### PBK reduces T-cell-mediated cytotoxicity dependent on CD276

**Fig. 6:**
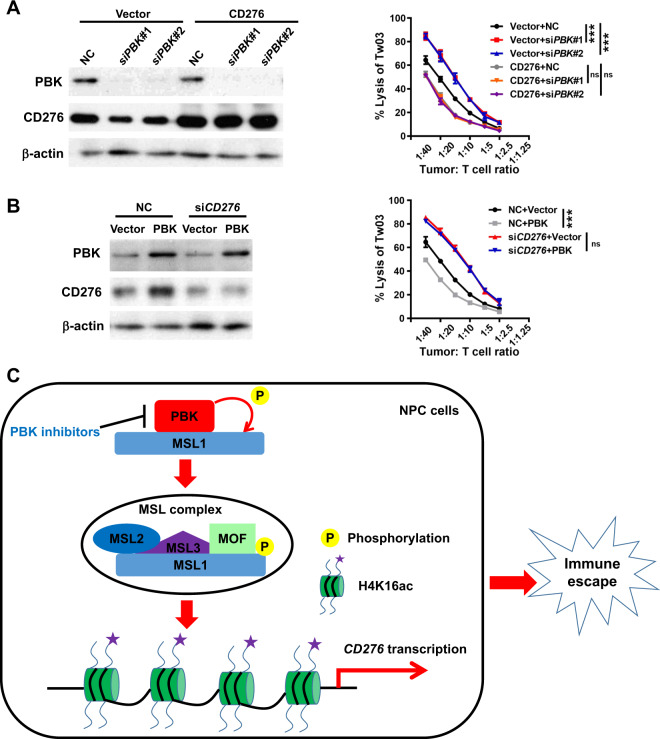
PBK modulates the T-cell-mediated killing in NPC cells mediated by CD276 in vitro. **A** TW03 cells were infected by lentiviruses carrying *CD276* coding region to establish the CD276-overexpression (OE) stable cell lines. Control and CD276-OE cells were transfected with two *PBK* siRNAs or NC (nonspecific siRNA) for 48 h. Then, the prepared tumor cells and activated T cells were cocultured at a ratio of 1:40–1:1.25 (tumor cells:T cells) in triplicates and the cytolysis of tumor cells was evaluated using the xCELLingence real-time cell analysis (RCTA) system. Left, immunoblotting analysis using indicated antibodies. Right, the cure displayed the percentage of lysis tumor cells at a ratio of 1:40–1:1.25 (tumor cells:T cells). Data shown are mean ± SD and the *P* values were calculated by one-way ANOVA. ***P < 0.01, ns (not significant) means *P* > 0.05. **B** TW03 cells were infected by lentiviruses carrying *PBK* coding region to establish the PBK-overexpression (OE) stable cell lines. Control and PBK-OE cells were transfected with one *CD276* siRNA or NC (nonspecific siRNA) for 48 h. Then, the cytotoxic T-lymphocyte assay was performed as mentioned above. Left, immunoblotting analysis using indicated antibodies. Right, the data shown are mean ± SD and the *P* values were calculated by Student’s *t* test (two-tailed). ***P < 0.01, ns (not significant) means *P* > 0.05. All the experiments were performed independently three times with similar results and the data are representative of three independent experiments. **C** Proposed model of the PBK/MSL1/CD276 signaling pathway that regulates immune invasion in NPC cells, as well as the intervention strategy.

In order to investigate whether the PBK/CD276 axis plays an important role in tumor immune evasion, we conducted the cytotoxic T-lymphocyte assay. Since the NPC cell lines used above did not express HLA-A2 that is important for tumor cell recognition by HLA-A2+ T cells, we selected another NPC cell line TW03 with HLA-A2 positive for the cytotoxic T-lymphocyte assay. The results showed that PBK knockdown significantly increased the percentage of lysis TW03 cells when the tumor cells were cocultured with CD3/CD28-activated human HLA-A2+ T lymphocytes, and this increase was markedly reversed by the recovery of CD276 (Fig. 6A). On the other hand, PBK overexpression significantly reduced the percentage of dead TW03 cells, and this decrease was markedly rescued by *CD276* knockdown (Fig. 6B). Collectively, these data confirm that PBK contributes to the limitation of T-lymphocyte attack on NPC cells dependent on CD276.

## Discussion

Herein, our findings provide new insight into understanding the potential role of PBK in tumor immunology. We demonstrate that *PBK* expression is correlated with infiltration levels of diverse immune cells in NPC. Specifically, the infiltration levels of tumor-promoting leukocytes, including neutrophil, monocyte, CD8-naive, nTreg, and Th2 cells, were positively correlated with *PBK* mRNA expression. Simultaneously, the infiltration levels of tumor-repressing leukocytes, including NK, B, Tfh, CD4+ T cells, and Th17, were negatively correlated with *PBK* mRNA expression. These correlation patterns are at least partly attributed to the transcription regulation of the *CD276* gene by PBK. Although the role of CD276 in antitumor immunity has been controversial with conflicting co-stimulatory and co-inhibitory functions, CD276 may exert a protumor effect on tumor progression in NPC based on the results that the expression of *CD276* is much higher in tumor samples than normal samples and is associated with the compromised immune microenvironment. Since several immunomodulatory agents, especially anti-PD1 and anti-PDL1 antibodies, have shown great promise in treating advanced NPC, the role of the PBK/CD276 axis in immunotherapy response is further to be elucidated.

PBK, a dual-specificity serine/threonine kinase, plays an important role in the activation of the Raf/MEK/ERK pathway to promote cell proliferation, colony formation, and cancer development. For example, PBK facilitates transformation by upregulating and activating ERK2 through an increase in AP-1 (c-Jun) transcriptional activity and increased tumorigenesis of HCT116 colorectal cancer^33,71^. PBK can also directly bind to and phosphorylate AP-1 (c-Jun) after solar ultraviolet exposure^72,73^. Besides a potential central player of the MAPK cascade mechanism, PBK binds with other proteins and promotes various cancer-related processes, such as PBK promotes tumor dissemination by direct phosphorylation of p53-related protein kinase (PRPK)^74^. PBK regulates autophagy by phosphorylating ULK1 and promotes glioma resistance to TMZ (temozolomide)^75^. Thus, more research is necessary to identify potential downstream targets of PBK. Herein, we identify MSL1 as a previous unknown substrate of PBK. Indeed, PBK phosphorylates MSL1 and promotes the formation of the MSL complex. However, the specific phosphorylation site(s) of MSL1 and target genes of the PBK/MSL1 axis are needed to be explored.

*CD276* mRNA is widely expressed in many tissues and notably aberrantly expressed in various types of cancer. However, compared to *PDL1*, the cellular mechanisms that regulate *CD276* expression in cancer cells are poorly understood. So far, only a few findings identified revealed that overexpression of CD276 in tumor tissues was highly correlated with decreased expression of several miRNAs as compared to normal tissues, suggesting that a microRNA regulatory mechanism is involved in its differential expression^76,77^. In this report, we first present an epigenetic modulation mechanism of *CD276* expression. Specifically, PBK phosphorylates MSL1 and promotes the enrichment of MSL complex on the promoter region of *CD276*, in turn leading to an increase in histone H4 Lys16 acetylation, thereby activating the transcription of *CD276* and suppressing the cytotoxic T-cell function (Fig. 6C). Further investigations need to be conducted to clarify which transcription factors are essential for *CD276* transcription.

In summary, what is noteworthy is that this is the first demonstration of PBK modulation of a co-inhibitory signal CD276 induction to escape from immunosurveillance in NPC. Our results highlight the potential clinical benefits of targeting both PBK and CD276 in NPC patients with a high risk of progression.

## Materials and methods

### Cell culture

The 293T cells and the human NPC cell lines, including CNE2, SUNE1, HK1, and CNE1, were maintained in Dulbecco’s modified Eagle’s medium supplemented with 10% FBS at 37 °C and 5% CO_2_. All the cell lines were tested for negative mycoplasma contamination.

### Sample collection

Fresh frozen tissues for qPCR analysis were resected from histopathologically and clinically diagnosed NPC patients. All samples were obtained from the tumor resource bank of Guangzhou Medical University Affiliated Cancer Hospital and Institute. All patients supplied informed consent.

### Plasmids and mutagenesis

Full-length human PBK cDNA was amplified and cloned into a pcDNA6B-his-myc vector (ECORI and XholI) or plenti-puro vector (ECORI and XholI). Full-length human MSL1 cDNA was amplified and cloned into pCMV-N-flag (BamHI and ECORI). ClonExpress II One Step Cloning Kit (Vazyme) was used according to the manufacturer’s instructions.

pcDNA6B-his-myc-PBK(T198A), pcDNA6B-his-myc-PBK(T198E), and pCMV-N-flag-MSL1(S/T > A) mutants were constructed using a QuikChange site-directed mutagenesis kit (Stratagene) according to the manufacturer’s instructions. Sequences of the constructs were all verified by Sanger sequencing.

The primers used are listed as follows:

1. PBK with pcDNA6B-his-myc vector:

forward: 5′-TAGTCCAGTGTGGTGGAATTCATGGAAGGGATCAGTAATTTCAAGA-3′

reverse: 5′-CGCGGGCCCTCTAGACTCGGACATCTGTTTCCAGAGCTT-3′

2. PBK with plenti-puro vector:

forward: 5′-CTACCGGACTCAGATCTCGAGATGGAAGGGATCAGTAATTTCAAGA-3′

reverse: 5′-GTCATCCTTGTAATCGAATTCGACATCTGTTTCCAGAGCTTCAACA-3′

3. MSL1 with pCMV-N-flag:

forward: 5′-GATAAGAGCCCGGGCGGATCCATGGAAAGGCGGATGCAGC-3′

reverse: 5′-TCTGTCGACGATATCGAATTCCTATTTCCTACACGTCCGGTGAG-3′

### PBK inhibitor

The PBK inhibitor OTS514 was purchased from Selleck (S7652) and dissolved in DMSO with a stocked concentration (50 mM). For the functional assays, NPC cell lines CNE2 and SUNE1 were treated with the OTS514 at two different concentrations, including 2.5 and 5 μM.

### siRNA transfection

The specific siRNAs and control nonspecific siRNA (NC) were obtained from Guangzhou RiboBio Co., Ltd. Cancer cells were counted and seeded into 6-well plates with 2 × 10^5^ cells/well. After 24 h, the cells were 30–40% confluent and transfected with siRNAs using RNAiMAX transfection reagents (Invitrogen) according to the manufacturer’s instructions. The cells were harvested for further experiments after 24 or 48 h. The siRNA targeting sequences were as follows:

si*PBK*#1: 5′-GAATATGGCAAGAGGGTTAAA-3′

si*PBK*#2: 5′-GGGAACTAGGCCACCTATTAA-3′

si*PBK*#3: 5′-GAAGTGTGGCTTGCGTAAATA-3′

si*MSL1*#1: 5′-CACCGGACGTGTAGGAAATAG-3′

si*MSL1*#1: 5′-ATGTTATCACTCGCTGATAAT-3′

si*CD276*: 5′-AAAGAAGATGATGGACAAG-3′.

### RNA extraction and qPCR

Total RNA was extracted using the TRIzol Reagent (Invitrogen) according to the manufacturer’s instructions. Reverse transcription was performed using a cDNA Synthesis Kit (Thermo, K1622). The quantitative real-time polymerase chain reaction was conducted using ChamQ SYBR Color qPCR Master Mix (Vazyme, Q411-02). The sequences of the PCR primers used for amplification were as follows:

*ACTB* forward, 5′-AAGGTCATCC CTGAGCT GAA-3′

*ACTB* reverse, 5′-TGACAAAGTG GTCGTTG AGG-3′

*PBK* forward, 5′-GCGGTGAGACTCTGGACTGA-3′

*PBK* reverse, 5′-CTGCATAAACGGAGAGGCCG-3′

*CD276* forward, 5′-GGAGAATGCAGGAGCTGAGG-3′

*CD276* reverse, 5′-GCCAGAGGGTAGGAGCTGTA-3′

*PDL1* forward, 5′-ACATGTCAGGCTGAGGGCTA-3′

*PDL1* reverse, 5′-TTGGTGGTGGTGGTCTTACC-3′

*PDL2* forward, 5′-CAGTGTTCTGCGCCTAAAGC-3′

*PDL2* reverse, 5′-GGTCCTGGGTTCCATCTGAC-3′

*IDO1* forward, 5′-CACTTTGCTAAAGGCGCTGT-3′

*IDO1* reverse, 5′-CCCTTCATACACCAGACCGT-3′

*IL10* forward, 5′-ATCAAGGCGCATGTGAACTC-3′

*IL10* reverse, 5′-CATTCTTCACCTGCTCCACGG-3′

### Immunoblotting

Protein lysates were electrophoresed by sodium dodecyl sulphate-polyacrylamide gel electrophoresis (SDS-PAGE) (10% gel) followed by iBlot transfer to polyvinylidene fluoride (Thermo Fisher Scientific). Membranes were blocked in 5% skim milk powder in TBS with 0.1% Tween-20 and then incubated overnight with primary antibody at 4 °C. The following primary antibodies were used: anti-PBK (PTG, 13739-1-AP), anti-CD276 (PTG, 14453-1-AP), anti-MSL1 (PTG, 24373-1-AP), anti-β-actin (PTG, 20536-1-AP), and anti-mouse and anti-rabbit peroxidase-conjugated secondary antibodies (CST, HAF007, and HAF008).

### Co-immunoprecipitation and mass spectrometry analysis

For co-immunoprecipitation (co-IP), treated cells were lysed in IP lysis buffer (50 mM Tri-Cl (pH 7.4), 150 mM NaCl, 0.5% NP-40, and 5 mM EDTA) supplemented with PMSF (Sigma, USA) and protease inhibitor cocktail (Roche). The cell lysates were incubated with the indicated antibodies or Flag-beads (Sigma) overnight at 4 °C and washed with lysis buffer 4 times. Proteins were eluted and detected using immunoblotting assays with mouse antibody to Flag (Sigma, M2), an antibody to MSL1, and other appropriate antibodies.

For mass spectrometry analysis, 293T cells were transfected with Flag-PBK or vector control for 48 h. Then treated cells were lysed in IP lysis buffer (50 mM Tri-cl (pH 7.4), 150 mM NaCl, 0.5% NP-40, and 5 mM EDTA) supplemented with PMSF (Sigma, USA) and protease inhibitor cocktail (Roche) and incubated with Flag-tagged affinity agarose beads (Sigma, M2) overnight at 4 °C. The beads were then washed 4 times with IP lysis buffer. The immunoprecipitates were eluted and separated by SDS-PAGE and then stained via silver staining, and the indicated bands were subjected to mass spectrometry analysis.

### qChIP assay

The ChIP assays were carried out with a ChIP assay kit (Upstate Biotechnology, Lake Placid, NY, USA) according to the manufacturer’s instructions. The cultured NPC cells were fixed with 1% formaldehyde for 10 min at room temperature. Then, the ultrasonic breaker was set to 10 s per ultrasonic cycle with 10-s intervals with 15 cycles. Subsequently, the fragments underwent centrifugation (30,237×*g*) at 4 °C (part of the DNA fragments were used as input). The supernatant was collected and added with IgG control (1 ug/ml) or indicated antibodies, including anti-MSL1 (1 ug/ml, Merckmillipore, ABE469), anti-MOF (1 µg/ml, PTG, 13842-1-AP), and anti-H4K16ac (1 µg/ml, CST, #13534), followed by incubation at 4 °C overnight. Protein Agarose/Sepharose was used to precipitate the endogenous DNA–protein complex. After a short period of centrifugation (1000×*g*), the supernatant was removed, and the nonspecific complex was washed. Following de-cross-linking at 65 °C overnight, DNA fragments were extracted, purified, and retrieved with phenol/chloroform. The enrichment of MSL1 or MOF or H4K16ac to *CD276* promoter region was detected via qPCR assay, and the specific primers of *CD276* promoter region were as follows:

5′-GGTGAATGCGCTTTTGCAGG-3′

5′-CAGAGTGGACACGCCTAACC-3′

### T-lymphocyte preparations

HLA-A2 expression of the T lymphocytes from healthy donors was screened via flow cytometry and only positive individuals subjected to leukapheresis collections. T lymphocytes were then isolated from peripheral blood lymphocytes by depletion of non-T lymphocytes using a Pan T Cell Isolation Kit (Cat# 130-096-535, Miltenyi Biotec). Isolated human T-lymphocyte cells were activated in a T-cell culture medium supplemented with Interleukin-2 (Cat# 200-02, Peprotech) in the pre-coated plate with anti-CD3 (Cat#300313, BioLegend) and anti-CD28 (Cat#302913, BioLegend).

### T-cell killing assay

The prepared tumor cells and activated T cells were cocultured at a ratio of 1:40–1:1.25 (tumor:T cells) in triplicate and the cytolysis of tumor cells was evaluated using the xCELLingence real-time cell analysis system.

### Data acquisition

Comprehensive immune cell abundance prediction is estimated via ImmuCellAI. The following RNA expression data of NPC samples were retrieved from GEO: GSE102349, GSE68799, GDS3341, GSE40290, and GSE53819.

### Statistical analysis

All the experiments were conducted by three biological replicates with similar results. Student’s *t* test was used to compare two independent groups of data. One-way analysis of variance was used to analyze the significance among multi groups. Pearson correlation analysis was performed as indicated. *P* value < 0.05 was considered statistically significant in all cases. Asterisk (*) means *P* < 0.05, asterisks (**) mean *P* < 0.01, and asterisks (***) mean *P* < 0.001. SEM means standard error of mean and SD means standard deviation. All statistical analyses were performed using the SPSS 16.0 or Graphpad 8.01.

### Supplementary information

SUPPLEMENTAL MATERIAL
