# Correction: PBK phosphorylates MSL1 to elicit epigenetic modulation of *CD276* in nasopharyngeal carcinoma

**DOI:** 10.1038/s41389-021-00325-y

**Published:** 2021-04-30

**Authors:** Meng-Yao Wang, Bin Qi, Fang Wang, Zhi-Rui Lin, Ming-Yi Li, Wen-Jing Yin, Yan-Yi Zhu, Lu He, Yi Yu, Fang Yang, Jin-Quan Liu, Dong-Ping Chen

**Affiliations:** 1 Department of Radiation Oncology, Affiliated Cancer Hospital and Institute of Guangzhou Medical University, 510245 Guangzhou, China; 2 State Key Laboratory of Oncology in South China, Collaborative Innovation Center for Cancer Medicine, Sun Yat-Sen University Cancer Center, 510245 Guangzhou, China

**Keywords:** Head and neck cancer, Tumour immunology

Correction to: *Oncogenesis*

10.1038/s41389-020-00293-9 published online 5 January 2021

The information that these authors contributed equally: Meng-Yao Wang, Bin Qi, Fang Wang, should include in the author information page.

The original article has been corrected.

