# Correction: PBK phosphorylates MSL1 to elicit epigenetic modulation of *CD276* in nasopharyngeal carcinoma

**DOI:** 10.1038/s41389-022-00399-2

**Published:** 2023-11-08

**Authors:** Meng-Yao Wang, Bin Qi, Fang Wang, Zhi-Rui Lin, Ming-Yi Li, Wen-Jing Yin, Yan-Yi Zhu, Lu He, Yi Yu, Fang Yang, Jin-Quan Liu, Dong-Ping Chen

**Affiliations:** 1 Department of Radiation Oncology, Affiliated Cancer Hospital and Institute of Guangzhou Medical University, 510245 Guangzhou, China; 2 State Key Laboratory of Oncology in South China, Collaborative Innovation Center for Cancer Medicine, Sun Yat-Sen University Cancer Center, 510245 Guangzhou, China

**Keywords:** Head and neck cancer, Tumour immunology

Correction to: *Oncogenesis* 10.1038/s41389-020-00293-9, published online 05 January 2021

There was a mistake in the western blots in Figure 2B, the corrected figure is given below.

The original article has been corrected.

Figure 2



