# Supplementary material for: PBK phosphorylates MSL1 to elicit epigenetic modulation of CD276 in nasopharyngeal carcinoma
Source: Oncogenesis. 2021 Jan 5;10(1):9. doi: 10.1038/s41389-020-00293-9 (PMC7801519; doi:10.1038/s41389-020-00293-9)
Supplement: Supplementary file 1 — SUPPLEMENTAL MATERIAL [file 41389_2020_293_MOESM1_ESM.doc]

**Supplementary data**


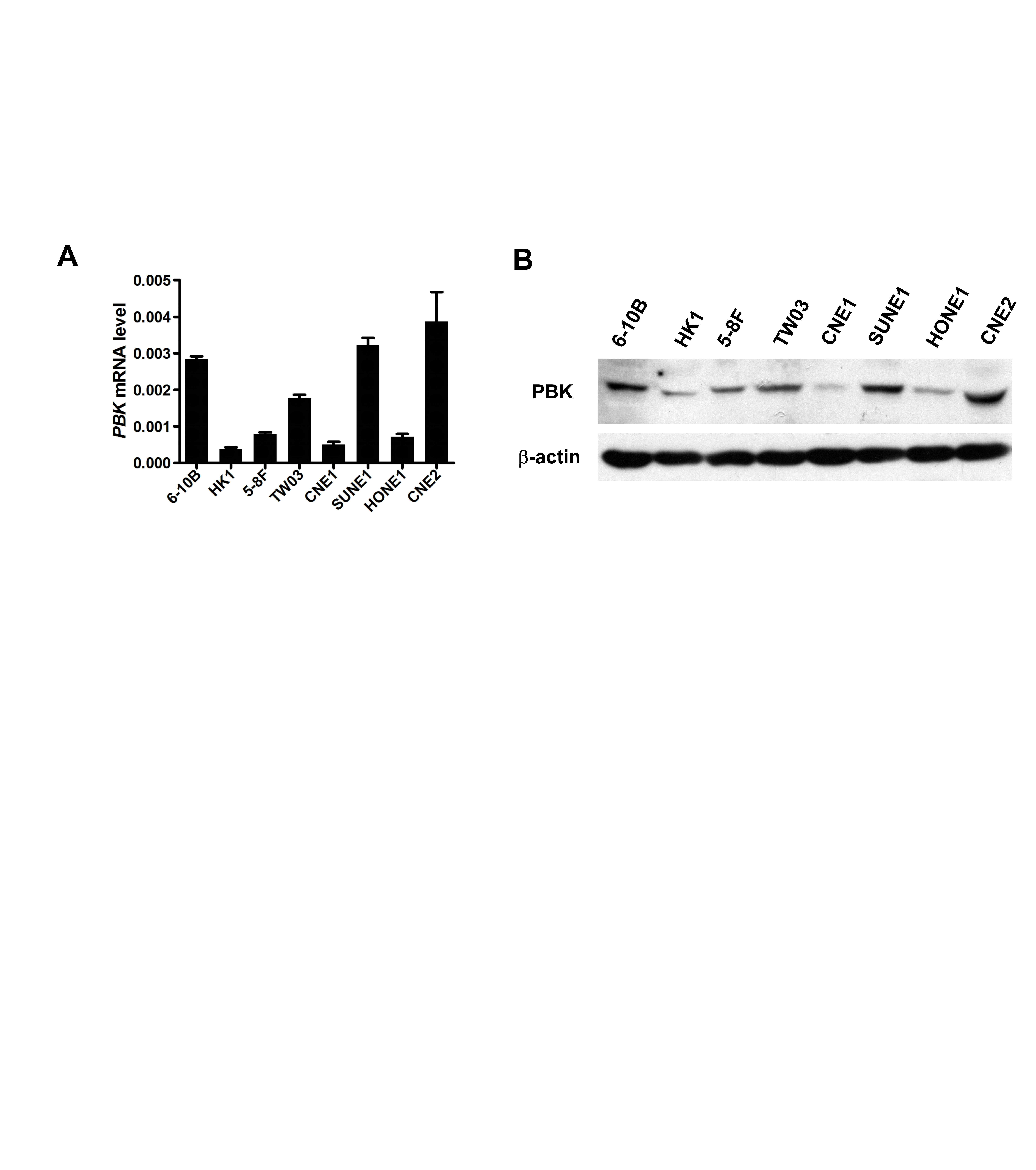


**Fig. S1 PBK expression analysis in NPC cell lines**

1. qPCR analysis of *PBK* mRNA level.
2. Immunoblotting analysis of PBK protein level.


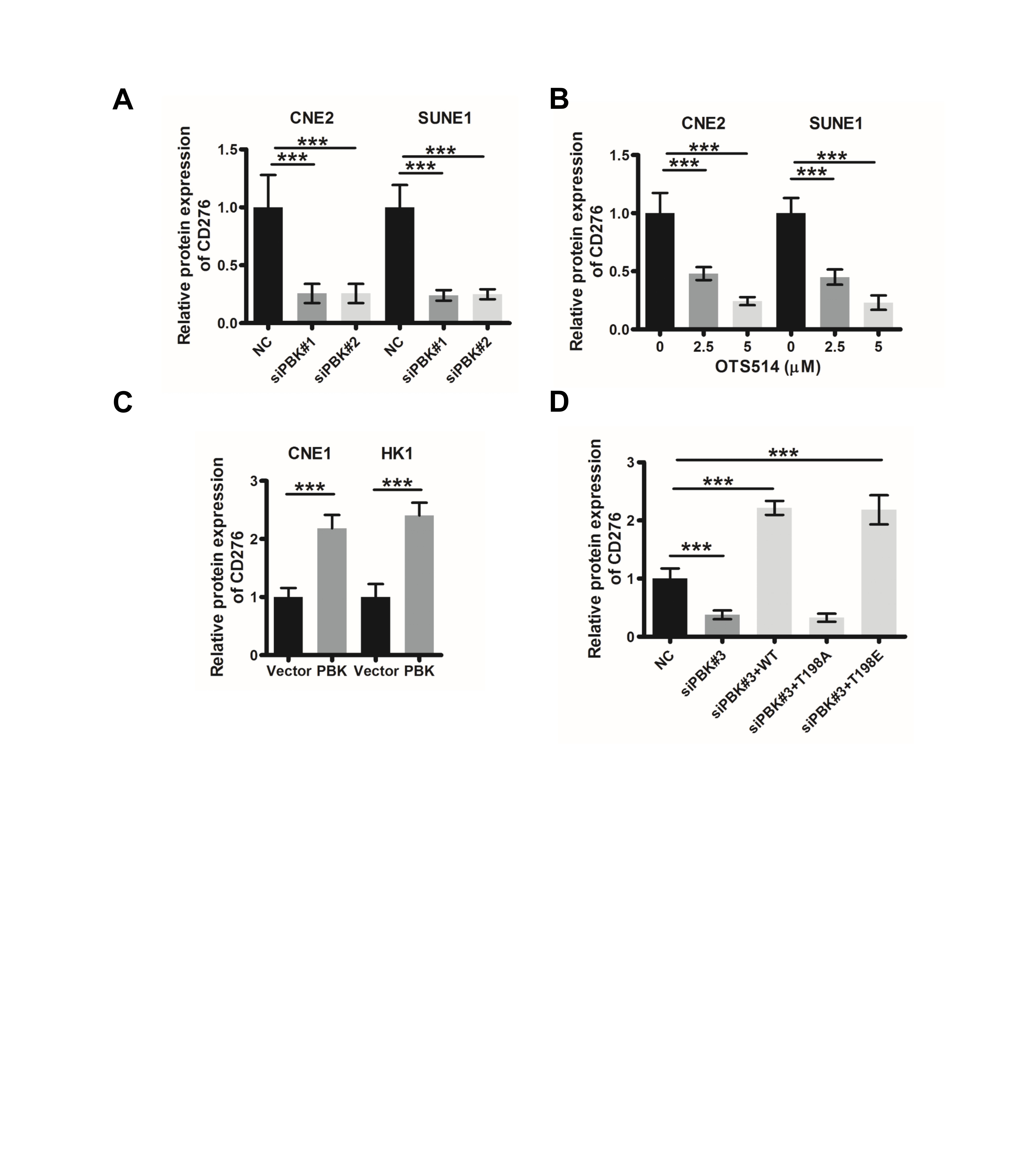


**Fig. S2 Quantitative analysis of WB in Figure 2**

1. Quantitative analysis of Figure 2B
2. Quantitative analysis of Figure 2C
3. Quantitative analysis of Figure 2D
4. Quantitative analysis of Figure 2E


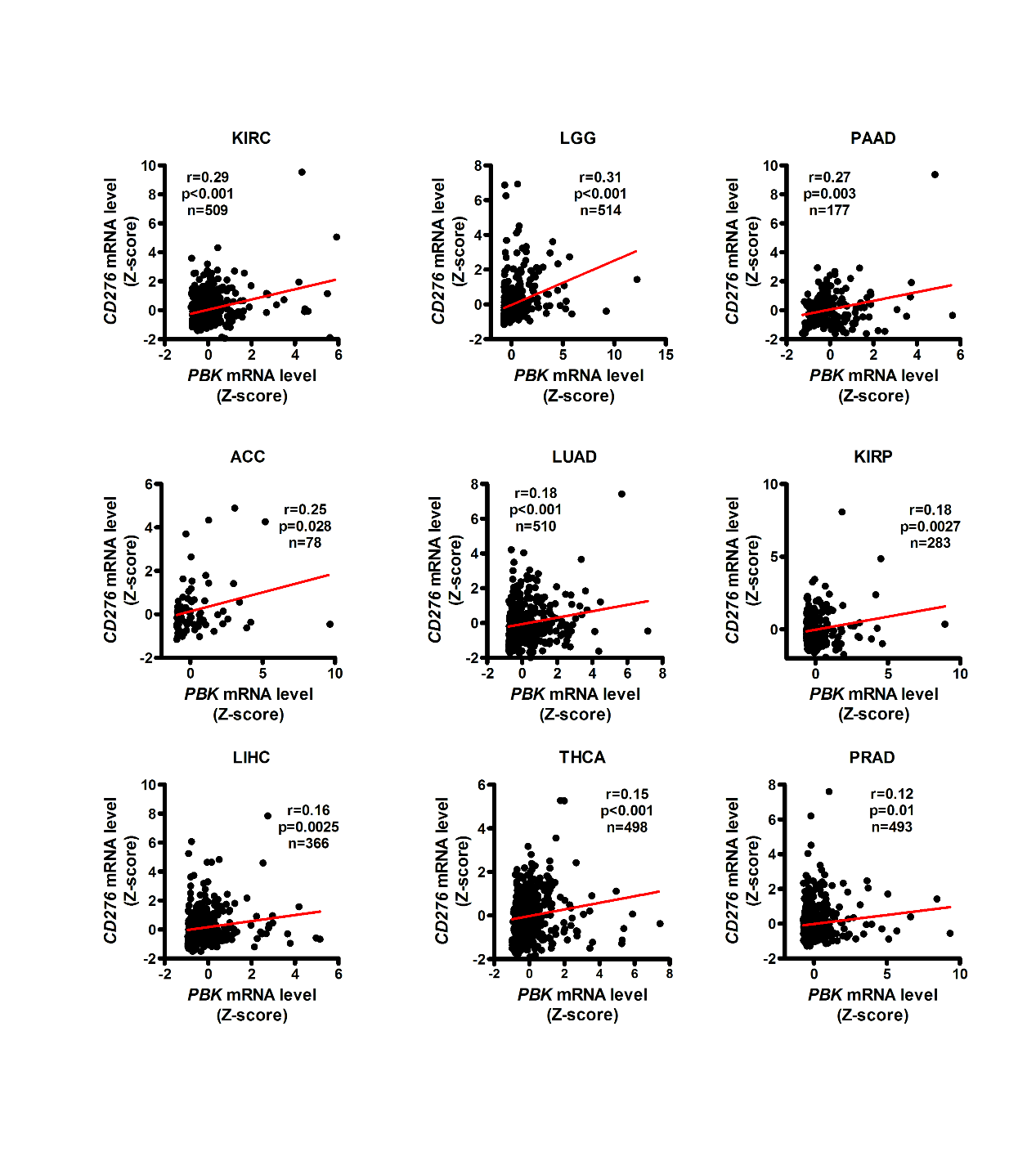


**Fig. S3 The mRNA level of *PBK* is positively correlated with *CD276* in pan-cancer**

adrenocortical carcinoma (ACC), brain lower grade glioma (LGG), kidney renal clear cell carcinoma (KIRC), kidney renal papillary cell carcinoma (KIRP), liver hepatocellular carcinoma (LIHC), lung adenocarcinoma (LUAD), pancreatic adenocarcinoma (PAAD), prostate adenocarcinoma (PRAD), thyroid carcinoma (THCA)


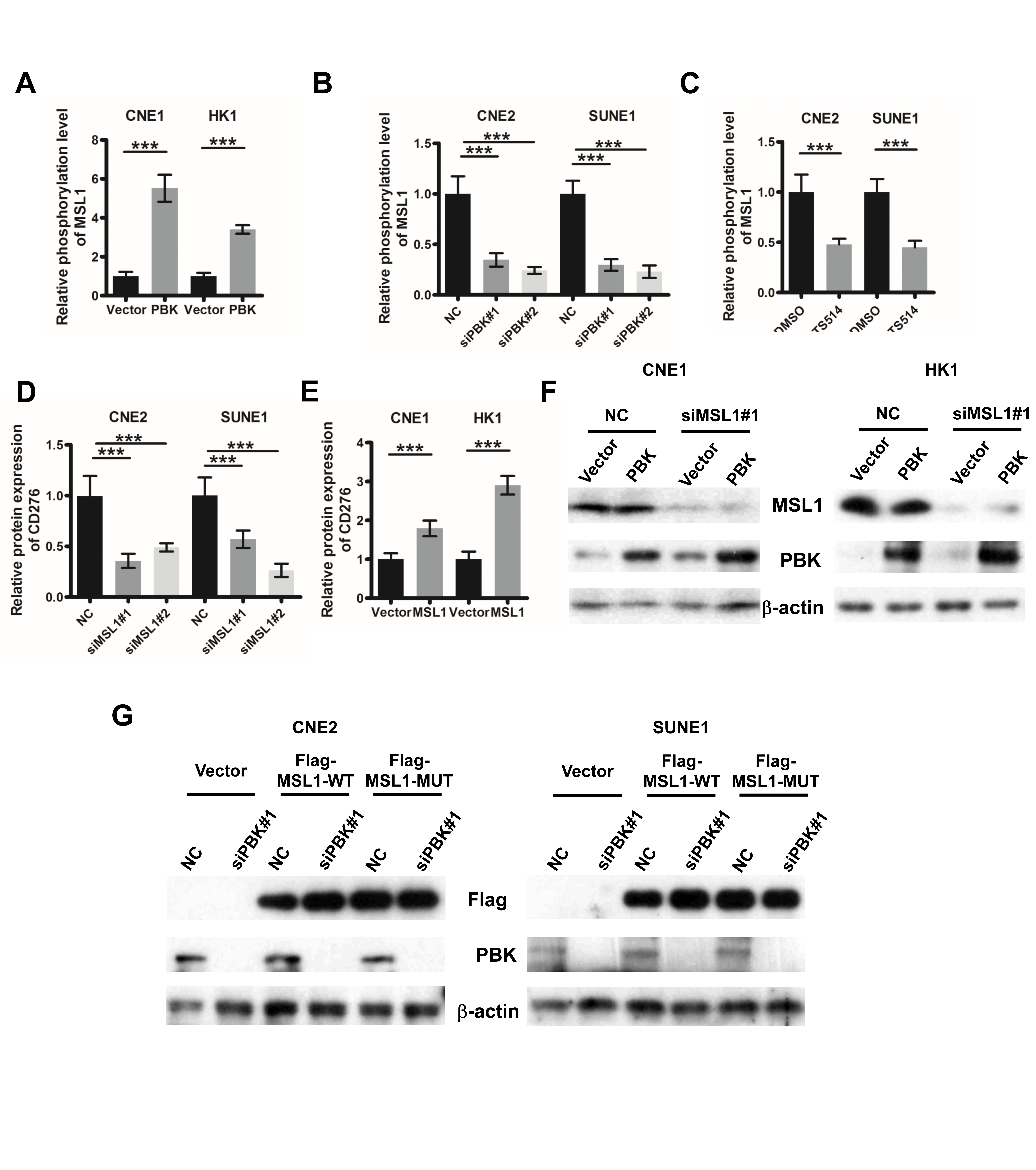


**Fig. S4** **Western blotting analysis of Figure 3**

1. Quantitative analysis of Figure 3D.
2. Quantitative analysis of Figure 3E.
3. Quantitative analysis of Figure 3F.
4. Quantitative analysis of Figure 3G.
5. Quantitative analysis of Figure 3H.
6. Western blotting analysis of Figure 3I.
7. Western blotting analysis of Figure 3J.

Left, CNE2 cells. Right, SUNE1 cells.


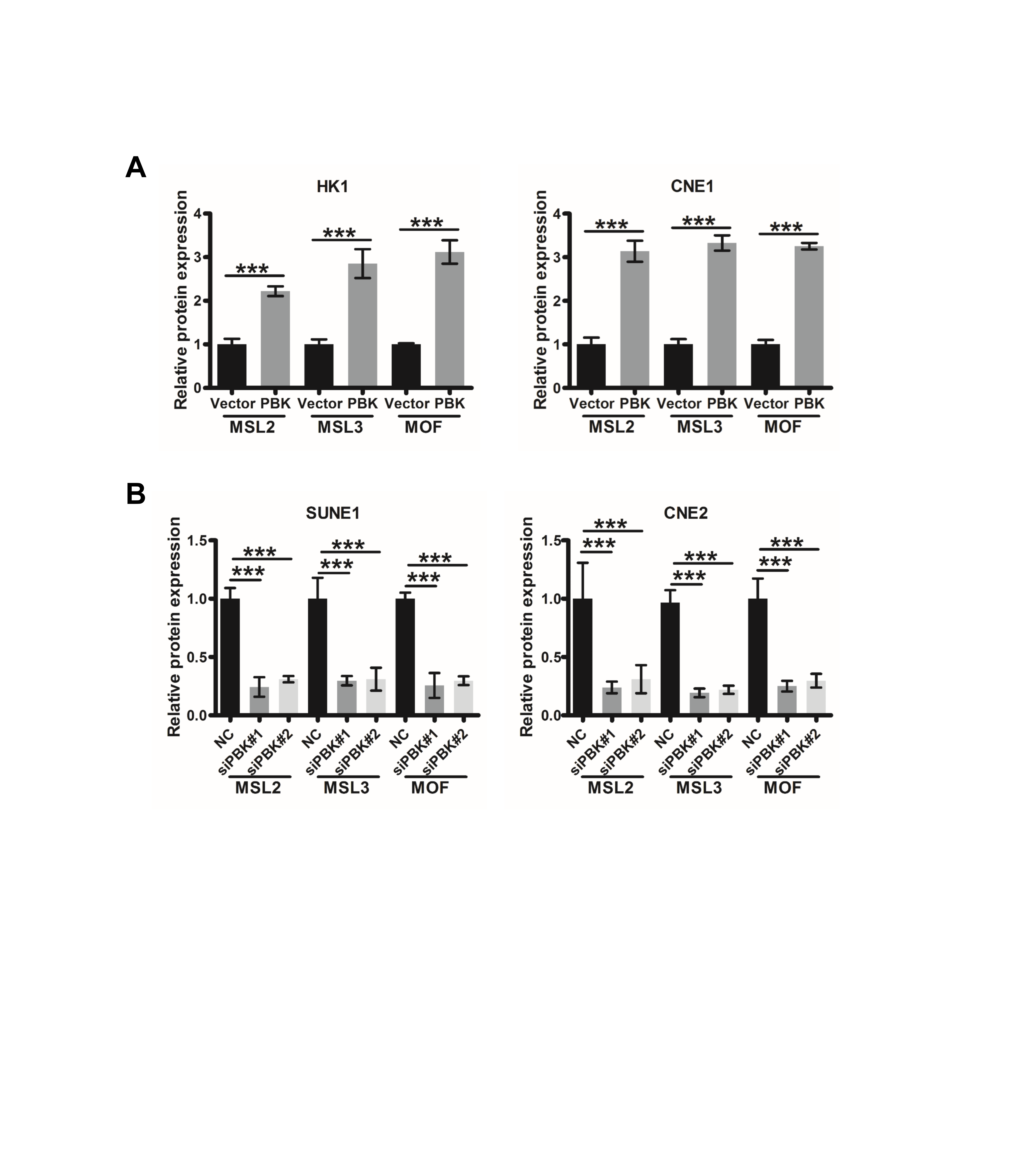


**Fig. S5 Quantitative analysis of WB in Figure 4**

1. Quantitative analysis of Figure 4A.
2. Quantitative analysis of Figure 4B.
